# Whole‐Body Magnetic Resonance Imaging Assessment of the Contributions of Adipose and Nonadipose Tissues to Cardiovascular Remodeling in Adolescents

**DOI:** 10.1161/JAHA.123.030221

**Published:** 2023-07-25

**Authors:** Jakob A. Hauser, Samuel J. Burden, Ajanthiha Karunakaran, Vivek Muthurangu, Andrew M. Taylor, Alexander Jones

**Affiliations:** ^1^ Centre for Translational Cardiovascular Imaging University College London London United Kingdom; ^2^ Department of Paediatrics University of Oxford, John Radcliffe Hospital Oxford United Kingdom; ^3^ Great Ormond Street Hospital for Children NHS Foundation Trust London United Kingdom; ^4^ Department of Women and Children’s Health King’s College London, St Thomas’ Hospital London United Kingdom

**Keywords:** anthropometry, cardiovascular system, heart, obesity, pediatric, Obesity, Pediatrics, Cardiovascular Disease

## Abstract

**Background:**

Greater body mass index is associated with cardiovascular remodeling in adolescents. However, body mass index cannot differentiate between adipose and nonadipose tissues. We examined how visceral and subcutaneous adipose tissue are linked with markers of early cardiovascular remodeling, independently from nonadipose tissue.

**Methods and Results:**

Whole‐body magnetic resonance imaging was done in 82 adolescents (39 overweight/obese; 36 female; median age, 16.3 [interquartile range, 14.4–18.1] years) to measure body composition and cardiovascular remodeling markers. Left ventricular diastolic function was assessed by echocardiography. Waist, waist:height ratio, and body mass index *z* scores were calculated. Residualized nonadipose tissue, subcutaneous adipose tissue, and visceral adipose tissue variables, uncorrelated with each other, were constructed using partial regression modeling to allow comparison of their individual contributions in a 3‐compartment body composition model. Cardiovascular variables mostly related to nonadipose rather than adipose tissue. Nonadipose tissue was correlated positively with left ventricular mass (*r*=0.81), end‐diastolic volume (*r*=0.70), stroke volume (*r*=0.64), left ventricular mass:end‐diastolic volume (*r*=0.37), and systolic blood pressure (*r*=0.35), and negatively with heart rate (*r*=−0.33) (all *P*<0.01). Subcutaneous adipose tissue was associated with worse left ventricular diastolic function (*r*=−0.42 to −0.48, *P*=0.0007–0.02) and higher heart rates (*r*=0.34, *P*=0.007) but linked with better systemic vascular resistance (*r*=−0.35, *P*=0.006). There were no significant relationships with visceral adipose tissue and no associations of any compartment with pulse wave velocity.

**Conclusions:**

Simple anthropometry does not reflect independent effects of nonadipose tissue and subcutaneous adipose tissue on the adolescent cardiovascular system. This could result in normal cardiovascular adaptations to growth being misinterpreted as pathological sequelae of excess adiposity in studies reliant on such measures.

**Nonstandard Abbreviations and Acronyms**
CO: cardiac output
E/A ratio: peak early‐to‐late Doppler mitral inflow velocity ratio
EDV: end‐diastolic volume
LVEDV: left ventricular end‐diastolic volume
rSAT: residualized subcutaneous adipose tissue
rVAT: residualized visceral adipose tissue
SAT: subcutaneous adipose tissue
SV: stroke volume
SVR: systemic vascular resistance
TAC: total arterial compliance
VAT: visceral adipose tissue

**Clinical Perspective**

**What Is New?**

- Whole‐body magnetic resonance imaging was used to determine the independent, relative contributions of adipose and nonadipose tissue to traditional markers of cardiovascular remodeling in adolescents.
- Our findings suggest that associations between anthropometrics and markers commonly associated with early cardiovascular remodeling are mediated predominantly by nonadipose tissue, to some extent by subcutaneous adipose tissue, and with little effect of visceral adipose tissue in this age group.
- Simple anthropometry does not reflect the independent effects of nonadipose tissue and adipose tissue on the adolescent cardiovascular system.

**What Are the Clinical Implications?**

- The associations of anthropometry with cardiovascular remodeling in adolescents could reflect early physiological adaption and not the adverse effects of raised adiposity, which might only emerge after chronic exposure to adiposity.
- This could result in normal cardiovascular adaptations to growth being misinterpreted as pathological sequelae of excess adiposity in studies reliant on such measures.
- It is important to consider the strong confounding effect of nonadipose tissue when interpreting associations between indirect anthropometric indicators of body composition, such as body mass index, and cardiovascular risk measures in the young.

Excess body fat (adiposity) in adulthood, especially visceral adipose tissue (VAT), and its associated risk factors such as hypertension, are significant determinants of cardiovascular disease (CVD) risk.^1^, ^2^, ^3^, ^4^, ^5^ Body size measures that are influenced by adipose and nonadipose tissue, such as body mass index (BMI), are typically used in large epidemiological studies to assess adiposity, which have shown that a higher BMI is linked with elevated CVD risk.^6^, ^7^, ^8^ In child and adolescent studies, an increased BMI and other measures of anthropometry have been associated with cardiovascular remodeling.^2^, ^9^, ^10^, ^11^, ^12^, ^13^, ^14^ This has been interpreted as key evidence that excess adiposity drives pathophysiological cardiovascular remodeling in this age group that underpins the associated CVD risk found in adults.^15^, ^16^, ^17^

Although simple and inexpensive, BMI is a poor measure of excess adiposity in the young^18^, ^19^, ^20^, ^21^ and cannot differentiate between adipose and nonadipose tissues.^22^, ^23^ This is especially critical in the pediatric population, where nonadipose tissue accrued through somatic growth correlates more strongly with BMI than adipose tissue does.^24^, ^25^ Thus, conclusions that excess adiposity is linked with adverse cardiovascular remodeling in the young could in some cases have been drawn due to a misinterpretation of why BMI was associated with those parameters. Indeed, a study of more than two thousand eight hundred 10‐year‐old children found that it was nonadipose tissue measured by dual‐energy x‐ray absorptiometry (DEXA) that was most strongly linked with early cardiac remodeling.^26^, ^27^

Although DEXA has been used extensively in the clinical and research setting because of its ease of use and relatively low cost, providing important insights into body composition and cardiovascular remodeling,^26^, ^27^ whole‐body magnetic resonance imaging (MRI) is seen as the research “gold standard.”^28^, ^29^ MRI provides true volumetric 3‐dimensional imaging, whereas DEXA produces 2‐dimensional images that are subsequently used to estimate tissue compartment volumes from anatomic models.^30^ To date, MRI use has been mainly limited due to the time and effort needed to manually segment anatomic regions, but with the development of efficient automated tools to segment 3‐dimensional images,^30^, ^31^, ^32^ regional tissue compartments can now be readily determined from whole‐body MRI. However, to date, there have been no studies to assess MRI‐derived whole‐body adiposity and how it is linked with cardiovascular remodeling in the young, independently from variations in nonadipose tissue. Controlling for the effect of nonadipose tissue is important, as cardiovascular remodeling can occur in response to the accrual of any tissue type.

As VAT has been identified as particularly influential on CVD risk in adults, compared with subcutaneous adipose tissue (SAT),^1^, ^3^, ^4^ we sought to determine how both VAT and SAT are linked with traditional markers of early cardiovascular remodeling, independently from nonadipose tissue and from each other, using MRI to assess both cardiovascular physiology and whole‐body composition. By comparing the patterns of relationship between markers of cardiovascular remodeling and BMI *z* score with those for MRI‐determined body composition, we sought to highlight which elements of body composition were likely to be responsible for the associations with anthropometric measures. Similar assessments were carried out for other anthropometric measures, such as waist circumference, which are sometimes considered to better reflect adiposity than BMI does.

## METHODS

The data that support the findings of this study are available from the corresponding author upon reasonable request.

### Study Population

Participants for this cross‐sectional study were recruited via newspaper advertisements and through an obesity clinic between September 2014 and May 2016. The newspaper advertisement invited interest from healthy teenagers aged 13 to 18 years, with adiposity ranging from normal weight to obesity. Thus, this population of recruits was not selected on any criterion other than age. Exclusion criteria were known hypertension or any other chronic conditions requiring hospital management, endocrine or congenital obesity, known or possible pregnancy, and MRI‐incompatible metal implants. The study complies with the Declaration of Helsinki, and ethical approval was obtained before recruitment (Research Ethics Committee London—Queen's Square, Ref 13/LO/1750). Informed consent was obtained from all participants or their parents/legal guardian, as applicable.

### Study Protocol

Study visits took place at 9:30 AM. Height and weight were recorded using calibrated devices, and BMI was calculated. Body surface area (BSA) was calculated using the Haycock formula.^33^ Age‐ and sex‐specific BMI *z* scores were used to define “normal weight” (>−2 and ≤1) and “overweight/obese” (>1) groups, based on the World Health Organization's normative data and *z* score definitions.^34^ Waist circumference was measured according to standard practice, and age‐ and sex‐specific waist and waist:height ratio *z* scores were calculated.^35^, ^36^ Blood pressure (BP) was measured as the average of 2 to 3 measures over 10 minutes, after participants had been resting for 15 (interquartile range, 12–17) minutes, using an oscillometric device (Datex Ohmeda, General Electric, Boston, MA).

### Echocardiography

Echocardiography was used to assess left ventricular (LV) diastolic function, following standard guidelines,^37^, ^38^ using a General Electric Vivid 7 (GE Healthcare, Chicago, IL). In the apical 4‐chamber view, the sample volume was placed between the tips of the mitral valve leaflets to measure the peak early‐to‐late Doppler mitral inflow velocity ratio. In the apical 4‐chamber view, the tissue Doppler imaging sample volume was placed at the lateral and septal basal regions of the LV myocardium to cover mitral annulus excursion in both systole and diastole to measure lateral and septal peak early‐to‐late diastolic tissue velocity ratios.

### MRI Protocol

MRI was performed on a 1.5 T Avanto system (Siemens, Berlin, Germany) using 2 spine coils and 2 body matrix coils. A vectorcardiogram was used for cardiac gating and heart rate monitoring. To obtain LV volumes, a stack of short axis cine images ranging from the apex to the atria was acquired using real‐time radial steady‐state free precession *k‐t* SENSE imaging during free breathing.^39^ Aortic blood flow was measured above the sinuses and at diaphragmatic level using cardiac‐gated spiral phase‐contrast MRI in a single breath‐hold of ≈11 seconds (spatial resolution 2.1×2.1 mm; temporal resolution 9.6 milliseconds).^40^ Body composition was determined from the neck to the knees using T2* IDEAL imaging as described previously (slice thickness, 10 mm; voxel size, 3×3×10 mm).^41^ Breath‐holding was used to prevent motion artifact in the thoracic and abdominal region. This technique provides water‐ and fat‐separated images and has been previously validated and shown to produce highly accurate measures of body fat distribution.^42^

### Cardiovascular MRI Post Processing

Cardiovascular MRI data were processed offline using custom plugins for OsiriX version 6.5.2 or later (Pixmeo, Bernex, Switzerland). Blood flow and cine data were analyzed by a single observer (J.A.H.). After initial automatic segmentation of phase‐contrast MRI data by an in‐house algorithm, contours were adjusted manually on each frame of the magnitude and phase images to calculate blood flow in the ascending and descending aorta. Pulse wave velocity was derived from aortic flow (*Q*) and area (A) curves as described previously.^43^ This method relies on the fact that pulse wave velocity = ∆*Q*/∆A in the reflection‐free part of early systole. Thus, pulse wave velocity was calculated by robust linear regression of the initial linear segment of unfiltered and noninterpolated data points in both the *Q* and *A* curves, and by division of the resulting gradient for *Q* by that of *A*. Total arterial compliance was estimated as total arterial compliance=stroke volume (SV)/(systolic−diastolic BP). Systemic vascular resistance (SVR) was calculated as SVR=mean BP/cardiac output (CO). LV end‐diastolic volume (LVEDV) and end‐systolic volumes were obtained by manual contouring of the endocardial LV borders in each slice in order to calculate SV (SV=LVEDV−LV end‐systolic volume). LV ejection fraction was calculated as ejection fraction=(SV/LVEDV)×100. LV myocardial volume was obtained by manual contouring of the epicardial LV border and subtraction of the endocardial from the epicardial volume. LV myocardial mass was calculated by multiplying myocardial volume by a density estimate of 1.05 g·mL^−1^.

### Adiposity MRI Post Processing

An in‐house algorithm initially contoured the T2* IDEAL data to produce separate contours for SAT and VAT for each slice. Briefly, a combined fat/water image was thresholded to remove the background, creating a mask that identified tissue. Then the fat‐fraction image was thresholded to identify fat tissue after application of that mask. Finally, the visceral compartment was identified by an algorithm that found the inner and outer boundaries of the subcutaneous fat by tracing radially from the edges of the image to the center of the body with the visceral compartment defined by this inner boundary. All data were then screened manually by a single observer (A.K.) and contours edited if necessary. The arms were excluded in all participants due to frequent signal loss at the periphery of the field in some participants. Within‐slice values were then summed to quantify total SAT and VAT volume. Nonadipose tissue was based on the residual volume after SAT and VAT were excluded. Quantification of body composition into 3 components was used to define the 3‐compartment model. This process took ≈30 to 40 minutes to complete per case, which is substantially less time than manually contouring SAT and VAT takes.

### Statistical Analysis

Statistical analyses were performed using Stata version 14.2 (StataCorp, College Station, TX). Continuous data were expressed as median (interquartile range). Associations between anthropometric markers of obesity (ie, BMI, waist circumference and waist:height ratio *z* scores) and cardiovascular measures were assessed by multiple linear regression analysis, adjusted for age and sex. This analysis was repeated for BMI *z* score adjusted for nonadipose tissue to isolate the effects of adipose tissue in these associations. This was done using partial regression analysis, taking the residual variability of BMI *z* score after nonadipose tissue was accounted for.^44^ Any nonparametrically distributed data were zero‐skewed log‐transformed by taking the natural log of the nonparametric variable after a correction factor had been applied (using the ‐*lnskew0*‐ function) before multiple linear regression.

To control for the strong intercorrelation between VAT, SAT, and nonadipose tissue, partial regression modeling was used to construct residualized whole‐body composition variables that had zero correlation with each other, allowing for their independent contribution to cardiovascular variables to be assessed. These were constructed as the residual variability of SAT after nonadipose tissue was accounted for (rSAT) and the residual variability of VAT after rSAT and nonadipose tissue were accounted for (rVAT). This sequence followed the lipid‐spillover hypothesis whereby lipids are typically stored first in SAT before being “spilled‐over” into VAT when the storage capacity of SAT is exceeded. Thus, our partial regression analyses resulted in 3 variables—nonadipose tissue, rSAT, and rVAT—that comprised the 3‐compartment model.

Multiple linear regression analyses were done to test the independent association of nonadipose tissue, rSAT, and rVAT with cardiovascular measures, adjusting for age and sex. These models included the 3 statistically independent tissue compartments that together described the total tissue volume of the body, thereby inherently adjusting the cardiovascular dependent variable for body size. Allometrically scaled cardiovascular variables (eg, those adjusted for BSA) were not used, as these would, by construction, impose a confounding correlation with the volumetric body composition measures. This was confirmed by multiple linear regression of both height and weight with BSA, adjusting for age and sex.

Similar models were done with the anthropometric measures to demonstrate the relative contribution of nonadipose tissue, rSAT, and rVAT to anthropometric measures in adolescents, including BSA.

To exclude the possibility that the recruits from obesity clinic introduced a bias into an otherwise unselected population of adolescents, we repeated our analyses, excluding these participants. Due to the multiple comparisons between cardiovascular structure/function measures and measures of adiposity, *P*<0.01 was considered statistically significant.

## RESULTS

### Population Characteristics

A comprehensive summary of the study population is presented in Table 1. Eighty‐two participants with median age 16.3 (interquartile range, 14.4–18.1) years completed the study, of whom 36 were female, and 39 had a BMI *z* score at or above the overweight/obese threshold (30 were classified as obese). Three participants were recruited from an obesity clinic, with the remainder coming from an unselected population via newspaper advert.

**Table 1:** Comparisons of Anthropometry, Body Composition, and Cardiovascular Measures Between Participants With Normal Weight and Those With Overweight/Obesity

	Normal weight	Overweight/obesity
No. (%)	43 (52)	39 (48)
Age, y	16.2 (14.3 to 18.1)	16.7 (14.4 to 18.3)
Sex, female, n, %	20 (47)	16 (41)
Height, cm	166 (160 to 174)	174 (166 to 179)
Weight, kg	55.3 (50.5 to 65.3)	87.1 (77.0 to 106.7)
Body surface area, m^2^	1.58 (1.51 to 1.88)	2.08 (1.89 to 2.34)
BMI, kg·m^−2^	20.4 (18.4 to 21.8)	29.6 (26.4 to 35.1)
BMI *z* score	−0.1 (−0.6 to 0.2)	2.4 (1.7 to 3.0)
Waist circumference	70.5 (65.5 to 75.8)	97.5 (84.0 to 107.5)
Waist circumference *z* score	−0.6 (−0.9 to 0.0)	1.6 (0.8 to 1.9)
Waist:height ratio	0.42 (0.40 to 0.45)	0.55 (0.49 to 0.63)
Waist:height ratio *z* score	−0.6 (−1.1 to 0.2)	1.3 (0.7 to 1.7)
NAT (L)*	27.5 (25.7 to 37.3)	40.0 (31.8 to 47.7)
SAT (L)*	9.6 (6.9 to 11.8)	30.5 (23.9 to 36.4)
VAT (L)*	0.5 (0.3 to 0.6)	1.4 (0.8 to 2.1)
Systolic BP, mm Hg	111 (106 to 117)	116 (112 to 127)
Diastolic BP, mm Hg	58 (55 to 62)	60 (55 to 67)
Mean BP, mm Hg	80 (77 to 84)	86 (79 to 89)
Pulse pressure, mm Hg	51 (47 to 59)	53 (50 to 63)
SVR (WU)	15 (13 to 17)	13 (12 to 14)
Cardiac output, L/min^−1^	5.6 (4.6 to 6.3)	6.3 (5.9 to 7.1)
Stroke volume, mL	82 (73 to 94)	93 (86 to 109)
Heart rate, bpm	69 (59 to 76)	67 (59 to 75)
LVEDV, mL	141 (124 to 158)	154 (132 to 178)
LVEF, %	63 (59 to 66)	62 (59 to 66)
LV myocardial mass, g	91 (81 to 115)	129 (104 to 153)
LV myocardial mass:LVEDV	0.74 (0.68 to 0.79)	0.82 (0.74 to 0.90)
E/A	1.85 (1.58 to 2.23)	1.66 (1.41 to 1.95)
Septal e′/a′	2.21 (1.92 to 2.77)	2.01 (1.57 to 2.30)
Lateral e′/a′	3.06 (2.15 to 3.46)	2.60 (2.34 to 3.19)
PWV_AAo_, m/s	4.1 (3.4 to 5.3)	4.3 (3.4 to 5.3)
PWV_DAo_, m/s	4.0 (3.1 to 5.1)	3.8 (3.1 to 5.0)
TAC, mL/mm Hg^−1^	1.6 (1.4 to 1.9)	1.7 (1.5 to 1.9)

AAo indicates ascending aorta; BMI, body mass index; BP, blood pressure; DAo, descending aorta; E/A, peak early‐to‐late Doppler mitral inflow velocity ratio; e′/a′, peak early‐to‐late diastolic tissue velocity; LV, left ventricular; LVEDV, left ventricular end‐diastolic volume; LVEF, left ventricular ejection fraction; NAT, nonadipose tissue; PWV, pulse wave velocity; SAT, subcutaneous adipose tissue; SV, stroke volume; SVR, systemic vascular resistance; TAC, total arterial compliance; VAT, visceral adipose tissue; and WU, wood units.

* Volumes based on neck‐to‐knee imaging, excluding arms.

Compared with the normal‐weight group, the overweight/obese group was taller; had higher BSA; and had higher BMI, waist circumference, waist:height ratio, and their respective *z* scores. Similarly, the overweight/obese group had more nonadipose tissue, SAT, and VAT.

### Correlation of Anthropometric Markers With Measures of Body Composition

In the 3‐compartment model (nonadipose tissue, rSAT, and rVAT), BSA and the anthropometry *z*‐scores (BMI, waist circumference, and waist:height) were correlated strongly and to a similar degree with both nonadipose tissue and rSAT (Table 2). There were trends toward associations between rVAT and the measures based on waist circumference. BSA demonstrated an almost perfect linear association with weight (*r*=0.99, *P*<0.0001) and a strong correlation with height (*r*=0.57, *P*<0.0001) after controlling for age and sex. No meaningful differences in the results were obtained after excluding the participants recruited from the obesity clinic.

**Table 2:** Standardized Independent Associations of Indirect Anthropometric Markers of Body Composition With 3 Statistically Independent Tissue Compartments Derived From Magnetic Resonance Measures of Tissue Volumes

	NAT*		rSAT*		rVAT*	
	*r*	*P*	*r*	*P*	*r*	*P*
BMI *z* score	0.83	<0.0001	0.89	<0.0001	−0.02	0.90
Waist *z* score	0.80	<0.0001	0.86	<0.0001	0.27	0.032
Waist:height *z* score	0.60	<0.0001	0.82	<0.0001	0.24	0.061
BSA	0.93	<0.0001	0.85	<0.0001	0.12	0.34

Associations are adjusted for age and sex. BMI indicates body mass index; BSA, body surface area; NAT, non‐adipose tissue; rSAT, residualized subcutaneous adipose tissue; and rVAT, residualized visceral adipose tissue.

* Volumes based on neck‐to‐knee imaging, excluding arms.

### Correlation of Anthropometric Markers With Cardiovascular Measures

BMI *z* score was correlated positively with LV mass, LV mass:EDV, and SV, and to a lesser extent with LVEDV and CO. There was a trend toward significance (*P*<0.05) with systolic BP, pulse pressure, and total arterial compliance (Table 3). There were inverse correlations with SVR and septal peak early‐to‐late diastolic tissue velocity ratio, and a trend toward an inverse correlation with the peak early‐to‐late Doppler mitral inflow velocity ratio. Adjustment of BMI *z* score for nonadipose tissue canceled out most of these associations but strengthened inverse correlations with the peak early‐to‐late Doppler mitral inflow velocity ratio (*r*=−0.42, *P*=0.003) and septal peak early‐to‐late diastolic tissue velocity ratio (*r*=−0.49, *P*=0.0006) and a positive correlation with diastolic BP (*r*=0.39, *P*=0.002). This adjustment also reversed the association of BMI *z* score with LVEDV, which then became a trend (*r*=−0.29, *P*=0.017). Associations with waist circumference and waist:height ratio *z* scores were similar to those with BMI *z* score. No meaningful differences in the results were obtained after exclusion of participants recruited from obesity clinic.

**Table 3:** The Pattern of Standardized Associations Between Cardiovascular Parameters and Traditional Anthropometry Compared With the Associations With 3 Statistically Independent Tissue Compartments

	BMI *z* score		Waist circumference *z* score		Waist:height ratio *z* score		3‐compartment model					
							NAT*		rSAT*		rVAT*	
	*r*	*P*	*r*	*P*	*r*	*P*	*r*	*P*	*r*	*P*	*r*	*P*
Systolic BP	0.29	0.015	0.27	0.026	0.23	0.059	0.35	0.005	−0.10	0.45	0.08	0.55
Diastolic BP	0.18	0.15	0.14	0.29	0.14	0.26	−0.22	0.091	0.42	0.0009	0.15	0.26
Mean BP	0.28	0.019	0.24	0.056	0.22	0.082	0.20	0.13	0.16	0.22	0.16	0.21
Pulse pressure	0.30	0.011	0.29	0.022	0.24	0.046	0.49	<0.0001	−0.29	0.024	0.02	0.89
SVR	−0.40	0.0005	−0.52	<0.0001	−0.41	0.0005	−0.27	0.032	−0.35	0.006	−0.01	0.95
CO	0.39	0.0004	0.52	<0.0001	0.42	0.0002	0.29	0.018	0.34	0.005	0.04	0.77
SV	0.48	<0.0001	0.50	<0.0001	0.41	0.0004	0.64	<0.0001	0.04	0.74	0.01	0.91
Heart rate	−0.07	0.56	0.07	0.54	0.06	0.62	−0.33	0.007	0.34	0.005	0.07	0.58
LVEDV	0.33	0.003	0.39	0.0007	0.26	0.024	0.70	<0.0001	−0.21	0.091	−0.12	0.32
LVEF	−0.04	0.70	−0.09	0.46	−0.05	0.64	−0.13	0.29	0.07	0.59	0.01	0.96
LV mass	0.58	<0.0001	0.56	<0.0001	0.46	<0.0001	0.81	<0.0001	−0.07	0.59	−0.13	0.30
LV mass:LVEDV	0.50	<0.0001	0.42	0.0002	0.40	0.0004	0.37	0.002	0.12	0.34	0.05	0.66
E/A	−0.29	0.029	−0.29	0.037	−0.28	0.046	0.10	0.51	−0.48	0.0007	−0.28	0.060
Septal e′/a′	−0.35	0.010	−0.30	0.040	−0.35	0.013	0.03	0.85	−0.45	0.002	−0.13	0.41
Lateral e′/a′	−0.06	0.68	−0.13	0.36	−0.16	0.26	0.03	0.85	−0.15	0.31	−0.29	0.048
PWV_AAo_	−0.01	0.92	0.00	0.99	−0.03	0.79	0.02	0.88	−0.10	0.42	0.00	0.99
PWV_DAo_	0.09	0.42	0.09	0.45	0.07	0.56	0.04	0.77	0.05	0.66	−0.10	0.43
TAC	0.28	0.020	0.29	0.018	0.25	0.040	0.10	0.43	0.30	0.021	−0.05	0.73

Associations are adjusted for age and sex. AAo indicates ascending aorta; BMI, body mass index; BP, blood pressure; CO, cardiac output; DAo, descending aorta; E/A, peak early‐to‐late Doppler mitral inflow velocity ratio; e′/a′, peak early‐to‐late diastolic tissue velocity; LVEF, left ventricular ejection fraction; LVEDV, left ventricular end‐diastolic volume; NAT, nonadipose tissue; PWV, pulse wave velocity; rSAT, residualized subcutaneous adipose tissue; rVAT, residualized visceral adipose tissue; SV, stroke volume; and TAC, total arterial compliance.

* Volumes based on neck‐to‐knee imaging, excluding arms.

### Correlation of Nonadipose Tissue, rSAT, and rVAT With Cardiovascular Measures

In the 3‐compartment model, nonadipose tissue explained the majority of the relationships of unadjusted BMI *z* score, waist circumference *z* score, and waist:height ratio *z* score with cardiovascular measures, but rSAT was responsible for some associations (Table 3). Nonadipose tissue was most strongly positively correlated with LV mass (*r*=0.81), LVEDV (*r*=0.70), and SV (*r*=0.64), independent of rSAT and rVAT. To a lesser extent, there were positive correlations of nonadipose tissue with systolic BP, pulse pressure, and LV mass:EDV, and a negative correlation with heart rate. rSAT was most strongly correlated with lower peak early‐to‐late Doppler mitral inflow velocity (*r*=−0.48) and septal peak early‐to‐late diastolic tissue velocity ratios (*r*=−0.45) and higher diastolic BP (*r*=0.42), independently of nonadipose tissue and rVAT. To a lesser extent, there were also independent positive correlations with CO and heart rate and a negative correlation with SVR. There were no independent cardiovascular associations with rVAT. No meaningful differences in the results were obtained after exclusion of participants recruited from obesity clinic.

## DISCUSSION

In this study, we have shown which tissue compartments drive the relationships between BMI and various measures that are commonly used to assess cardiovascular risk and dysfunction. We presented our principal results as standardized regression coefficients to highlight the patterns of associations with different tissues in the 3‐compartment model and facilitate their comparison. As the 3‐compartment model accounts for the strong intercorrelation between tissue compartments, this comparison revealed that some correlations of anthropometry with cardiovascular remodeling measures were due to underlying relationships with nonadipose tissue, some were explained by rSAT, and some were due to the influence of both nonadipose tissue and rSAT, not always in the same direction.

Our results suggest that previous studies that used BMI or similar anthropometric measures to support generalized statements that it is adolescent adiposity that drives early cardiovascular remodeling or those that apparently assumed that all risk factor changes associated with greater BMI represented pathology^15^, ^16^, ^17^ could have misinterpreted their results due to the important confounding effect of nonadipose tissue on anthropometric measures of adiposity. For example, a progressive increase in BP as children age is a well‐known phenomenon and is routinely interpreted as an expected consequence of normal growth, which is, essentially, accrual of nonadipose tissue. Such an association would not typically be regarded as being reflective of a pathological process and could have a different impact on long‐term risk in comparison with a pathological process that also increases BP. Associations between measures of cardiovascular remodeling and BMI can reflect effects of nonadipose tissue and should, therefore, be interpreted cautiously in young people whose stage of childhood growth can be relatively advanced or retarded for their age. Importantly, our results suggest that alternative indirect anthropometric estimates of body composition, such as waist circumference or waist:height ratio, do not overcome this limitation.

Some of the most studied markers of early cardiovascular remodeling are those related to LV hypertrophy. Past research has consistently shown that increased BMI is associated with early signs of concentric LV remodeling.^2^, ^9^, ^10^, ^11^, ^12^, ^13^ Indeed, in this study, BMI *z* score was associated with an increase in LV myocardial mass, EDV, and myocardial mass:EDV ratio. This has led some studies to conclude that it is excess adiposity, assessed by BMI, that drives early cardiovascular remodeling.^15^, ^16^ However, increased BMI and other anthropometric measures reflect growth status and nonadipose tissue just as much they do adipose tissue,^24^, ^25^ raising concerns about tissue specificity when interpreting correlations with BMI. This was evident in the current study, where BMI *z* score was strongly correlated with both nonadipose tissue and rSAT. In fact, once BMI was adjusted for nonadipose tissue, the relationships of BMI *z* score with LV concentric remodeling markers were removed or even reversed. In our 3‐compartment model, only nonadipose tissue was linked to LV structural remodeling measures, supporting earlier findings from DEXA studies.^26^, ^27^ Our study also adds to the small MRI study by Dias et al^45^ (n=20 [obese n=9]), whereby abdominal adiposity was linked with measures of concentric hypertrophic remodeling, but these relationships were subsequently removed when indexed to DEXA fat‐free mass. Thus, it seems that it is nonadipose tissue that drives the early concentric LV remodeling that has been described in adolescent obesity.

Increased systolic BP, particularly in the context of increased vascular stiffness, is a prominent risk factor for CVD.^46^ In line with previous studies, we show that greater BMI *z* score is associated with higher systolic BP. However, higher systolic BP was not independently associated with adipose tissue in our study but was instead associated with nonadipose tissue. We can see from the results in Table 3 that the higher systolic BP is likely to be a result of higher CO, driven by greater SV linked to greater LV preload (LVEDV), all of which might be considered normal adaptations to having a larger body, irrespective of tissue type. To maintain physiological homeostasis, lower vascular resistance would be needed to accommodate greater CO without marked elevations in BP. Indeed, there was a trend toward lower SVR in those with greater nonadipose tissue. Interestingly, higher rSAT was also independently associated with lower SVR and a trend toward higher total arterial compliance. This supports suggestions that an initial protective mechanism exists in adolescent overweight/obesity to minimize vascular wall stress by adaptive vasodilation.^47^, ^48^ Together, these findings suggest that early remodeling in adolescents could reflect normal physiology. Furthermore, the lack of correlations between any tissue compartment and pulse wave velocity suggests that pathological vascular stiffening is not yet present in this age group, irrespective of body type. This is consistent with suggestions that many years of sustained exposure to adiposity may be needed before a demonstrable impact on vascular stiffness becomes apparent.^47^, ^48^

Although LV structural remodeling may reflect normal physiology, there may also be pathophysiological functional adaptations to excess adipose tissue. We found that BMI *z* score was correlated with markers of reduced LV diastolic function, especially with measures of reduced LV septal myocardial motion, supporting findings from our previous meta‐analysis.^14^ This builds on earlier work that did not account for the potential confounding effect of nonadipose tissue when investigating myocardial function.^45^ In the 3‐compartment model, we found that this was driven by rSAT, and to a lesser extent by rVAT. It could be that LV diastolic function is more susceptible to insult from excess adipose tissue than other cardiovascular risk markers, suggesting a role for diastolic function measures as some of the earliest markers of obesity‐related cardiac dysfunction. Mechanisms of adiposity‐driven LV diastolic dysfunction, such as reduced myocardial energetics,^49^, ^50^ are worth further exploration in this population to help understand the potential pathophysiology in adolescents with overweight/obesity.

In adolescence, associations between BMI and markers commonly used to assess cardiovascular risk, such as LV myocardial mass and BP, appear to reflect physiological adaptation to somatic growth, rather than adiposity. Although children with overweight/obesity arrive at similar adult heights to those with normal weight, they often exhibit accelerated skeletal maturation before this.^51^, ^52^ This was confirmed in our study, where adolescents with overweight/obesity were taller and had more nonadipose tissue. Normal growth in children and adolescents is associated with upward trends of LV myocardial mass, LV relative wall thickness, and increases in BP due to normal nonadipose tissue accrual.^53^, ^54^ As accelerated skeletal maturation is typically found in child and adolescent obesity, perhaps the associations of LV concentric remodeling with nonadipose tissue reflect accelerated yet normal physiological adaptations to growth, rather than pathological processes. It could be that early cardiovascular remodeling in adolescence begins as physiological adaptations but becomes pathophysiological in adulthood after many years of sustained exposure to excess adiposity.

Although cardiovascular remodeling may initially be normal, some have suggested that adolescent obesity may be linked with markers of pathophysiological remodeling. Statistical‐shape modeling of >2600 left ventricles in children identified that an increase in BSA was associated with an increased sphericity of the left ventricle.^12^ As discussed by the authors, increased sphericity of the LV is linked with pathophysiological LV remodeling in adults, potentially indicating an early pathophysiological remodeling pattern in children with obesity.^12^ However, as we demonstrated in this study, BSA is profoundly related to both nonadipose tissue and rSAT, being almost perfectly correlated with weight in our studied age group. Thus, it may not be possible to draw reliable conclusions about whether it is adipose tissue or nonadipose tissue that is responsible for such remodeling, based on an analysis of associations with BSA. Longitudinal studies may be needed to assess whether increased LV sphericity in the young is linked with poor cardiovascular health in later life, independently from adult risk factors and remodeling, before we can safely conclude that such changes in the young are likely to be pathophysiological.

As BMI reflects both adiposity and nonadipose tissue, false‐negative findings can result from opposing effects of each, potentially masking any true adverse effects of excess adipose tissue. For example, a high resting heart rate has been linked to adverse CVD outcomes.^55^ In our results, BMI *z* score was not associated with heart rate, but there were similar but opposite associations of heart rate with nonadipose tissue, which was linked to lower heart rate, and rSAT, which was linked to higher heart rate. Similarly, there were opposite associations of nonadipose tissue and rSAT with LVEDV (albeit with the latter not achieving statistical significance [*P*=0.091]). Thus, when interpreting nonsignificant or weak associations with BMI *z* score, it is important to consider the possibility of conflicting relationships between tissue compartments, as early pathophysiological adaptations to excess adipose tissue could be masked.

Nonhemodynamic factors such as hyperinsulinemia and inflammation have been identified as promotors of LV hypertrophy in obesity.^56^ Excess adipose tissue, particularly VAT, is strongly linked to such factors.^5^, ^23^ While this study was not designed to assess metabolic or inflammatory promotors of LV hypertrophy, we demonstrated that excess rVAT was not correlated with a panel of cardiovascular markers, apart from weak correlations with LV diastolic function measures. We interpret this as evidence that our adolescent participants had either not yet accrued sufficient excess VAT for there to be such adverse effects or that the proinflammatory and metabolic effects of VAT may be cumulative and require more time to have a demonstrable adverse impact on the cardiovascular system.

### Strengths and Limitations

One strength of this study was the use of whole‐body MRI, the research gold standard for noninvasive assessment of body composition, which allows reliable and accurate quantification of tissue compartments in different topographic locations. Furthermore, we used partial regression analyses to construct body‐composition variables that were uncorrelated with each other, allowing for their individual contributions to cardiovascular remodeling markers to be assessed. We used cardiovascular measures that had not been standardized for BSA and justified this by demonstrating the strong confounding associations that such adjustment would impose in the context of body composition analysis due to very strong associations of BSA with weight, height, nonadipose tissue, and rSAT. We studied an unselected population except for 3 recruits from an obesity clinic, but importantly, sensitivity analyses showed that our results were not meaningfully altered by their inclusion. As this study was cross‐sectional in design, the directions of the associations we demonstrated, if causal, could not be determined.

## CONCLUSIONS

Associations between BMI and markers commonly associated with early cardiovascular remodeling are mediated predominantly by nonadipose tissue, to some extent by rSAT, and with little effect of rVAT in adolescents. Many of these associations could reflect early physiological adaption and not the adverse effects of raised adiposity, which might emerge only after chronic exposure to adiposity. It is important to consider the strong confounding effect of nonadipose tissue when interpreting associations between indirect anthropometric indicators of body composition, such as BMI, and cardiovascular risk measures in the young. Longitudinal and intervention studies are needed to confirm these independent early remodeling patterns of nonadipose tissue, rSAT, and rVAT on the adolescent cardiovascular system and to determine whether these reflect pathophysiological or normal physiological processes.

## Sources of Funding

Dr Jones is supported by a British Heart Foundation Intermediate Clinical Research Fellowship (FS/18/22/33479). Dr Hauser was funded as part of the Framework Package‐7 project MD‐Paedigree of the European Commission (grant to Dr Taylor; contract no. 600932). Dr Burden is currently funded as part of a British Heart Foundation Special Project held at King's College London (SP/F/21/150013).

## Disclosures

Dr Hauser is currently an employee of Janssen Pharmaceutical Companies of Johnson & Johnson and was an employee of University College London at the time this work was conducted. The remaining authors have no disclosures to report.
